# Active Pharmaceutical Ingredient-Ionic Liquids (API-ILs): Nanostructure of the Glassy State Studied by Electron Paramagnetic Resonance Spectroscopy

**DOI:** 10.3390/molecules27165117

**Published:** 2022-08-11

**Authors:** Olga D. Bakulina, Mikhail Yu. Ivanov, Dmitriy V. Alimov, Sergey A. Prikhod’ko, Nicolay Yu. Adonin, Matvey V. Fedin

**Affiliations:** 1 International Tomography Center SB RAS, Institutskaya Street 3a, 630090 Novosibirsk, Russia; 2 Physics Department, Novosibirsk State University, Pirogova Street 2, 630090 Novosibirsk, Russia; 3 Boreskov Institute of Catalysis SB RAS, Lavrentiev Avenue 5, 630090 Novosibirsk, Russia

**Keywords:** EPR, ionic liquids, pharmaceuticals, nanostructuring, spin probes

## Abstract

Active Pharmaceutical Ingredient-Ionic Liquids (API-ILs) draw increasing interest as a particular class of ILs that possess unusual physicochemical properties along with simultaneous potentials for pharmaceutical applications. Although nanostructuring phenomena were actively investigated in common ILs, their studies in API-ILs are scarce so far. In this work, using the complex methodology of Electron Paramagnetic Resonance (EPR) and dissolved spin probes, we investigate nanostructuring phenomena in a series of API-ILs: [C_n_mim][Ibu], [C_n_mim][Gly], and [C_n_mim][Sal] with *n* = 2, 4, and 6, respectively. We reveal similar trends for API-ILs and common ILs, as well as peculiarities inherent to the studied API-ILs. Unusual behavior observed for [C_n_mim][Ibu] has been assigned to the presence of a non-polar fragment in the [Ibu]^−^ anion, which leads to the formation of more complex nanostructures around the radical compared to common ILs. Understanding general trends in the formation of such self-organized molecular structures is of fundamental interest and importance for applying API-ILs.

## 1. Introduction

Ionic liquids (ILs) are a unique class of organic salts that are liquid at temperatures below 100 °C and exhibit exceptional physicochemical properties, such as tunable melting points, nonflammability, nonvolatility [1], and nano- and micro-heterogeneity [2,3,4,5,6]. ILs are actively used in catalysis, fuel science, biotechnology, biomedicine, etc. [7,8,9,10,11,12,13,14]. In recent years, the use of ILs in the fields of medicine and pharmaceutics has expanded significantly. Converting active pharmaceutical ingredients into IL form (yielding API-IL) greatly improves solubility, dissolution rates, bioavailability, and stability such that the dual functionality of using active anions and cations can be pursued [15,16,17,18,19,20,21].

There are three interdependent conditions that should be simultaneously satisfied for the successful pharmaceutical application of any substance: molecular mobility (solubility), chemical stability, and selective activity [19]. It is well known that the majority of pharmaceutical products used worldwide are in an amorphous state, which is prone to spontaneous conversions into a crystal state. The physical stability of amorphous compounds is closely related to molecular mobility and relaxation properties in supercooled and glassy states. For this reason, knowledge of glass transition properties is crucial for revealing possible pharmaceutical applications of compounds, even if glass transitions are well below practical-application temperatures [22,23].

The characterization and structural dynamics of API-IL compounds are tightly bound to the glassy state properties of corresponding ILs. Since the stability of pharmaceuticals depends on its mobility in the glassy state, the majority of the related articles focused on the relaxation behavior [22,24]. For example, the API-IL based on water-insoluble ibuprofen and the [C_2_OHmim]^+^ cation possesses a water solubility of 10^5^ times higher than that of the initial ibuprofen [25]. It was shown that the *T*_g_ of [C_2_OHmim][Ibu] gradually increases with the ionic conductivity of the medium through dehydration [26]. [C_2_OHmim][Ibu] demonstrates a complex relaxation behavior in glassy and supercooled states. The research on several ibuprofenate API-ILs revealed a glass-to-metastable liquid phase transition with the freezing of orientational polarization at ~1.3 *T*_g_*,* which differs from common molecular liquids in which the freezing of polarization above *T*_g_ does not occur [27]. Thus, API-ILs represent an interesting group of ILs with different physicochemical behaviors and potentially different nanostructuring at the molecular level, showing great promise for biomedical and pharmaceutical applications.

In our previous research, we developed a targeted methodology of Electron Paramagnetic Resonance (EPR) for investigating nanostructuring phenomena in various ILs [28,29,30,31,32]. In particular, it has been shown that the continuous wave (CW) and pulse EPR data fruitfully complement each other and allow the structural dynamics of glassy ILs to be revealed [28,29,30]. Since an overwhelming number of ILs are diamagnetic, spin probes (stable nitroxide or trityl radicals) were dissolved in the studied media in trace concentrations and monitored using EPR. We showed that the use of TEMPO-D_18_ for CW–EPR studies and spirocyclohexane-substituted nitroxide N1 for pulse EPR studies (vide infra) constitute the best combination for obtaining complementary information [29,30].

**Figure 1:**
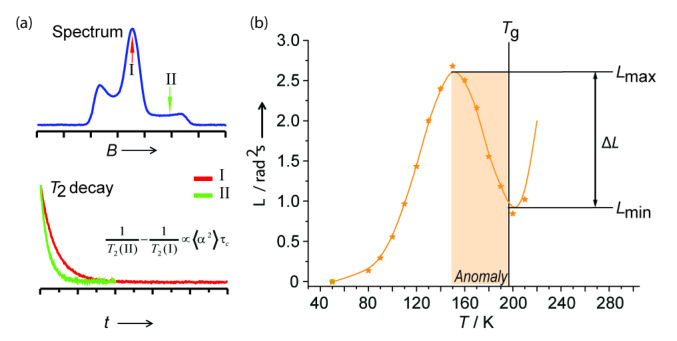
(**a**) The two-pulse electron spin-echo detected spectrum of the nitroxide radical (top). The *T*_2_ decay is measured by an incremental delay, τ, in the π/2-τ-π-τ- echo sequence at two spectral positions, I and II (bottom). Corresponding *T*_2_ times are obtained by a monoexponential analysis. (**b**) Schematic representation of the temperature dependence of the motional parameter, *L,* for thenitroxide radical in ILs and other organic glasses, showing a low-temperature region with negligible librations (*T* < 60 K) and then their onset, the linear region of effective librations (*T*~100–150 K), the anomaly region (*T*~150–200 K), and the transition into a liquid state with a steep increase in molecular motions at *T*_g_. This *L*(*T*) dependence was obtained for the spin probe N1 (Scheme 1) that was dissolved in the ionic liquid, [BuPy][BF_4_] (more details in [30]).

In addition to characterizing the local rigidity/stiffness/fragility of ILs, the developed methodology also reveals so-called structural anomalies in ILs [29,30,32,33,34]. In such anomalous temperatures regions near the glass transition, the molecular mobility decreases upon a temperature increase, which opposes general expectation for most media and materials [29,35]. To study the molecular mobility in IL glasses, we employed an EPR detection of stochastic molecular librations of a spin probe. In this method, transverse relaxation time, *T*_2_, was measured at two spectral positions (conditionally assigned as (I) and (II) in Figure 1a), which allows one to calculate the so-called librational parameter, *L*. The value of *L* is proportional to the mean squared amplitude of the librational motion around a fixed axis ⟨α^2^⟩ and the correlation time of such motion, τ_c_: *L* ≈ (1/*T*_2_^(II)^ − 1/*T*_2_^(I)^) = 10^11^⟨α^2^⟩τ_c_ [36,37,38,39,40]. The consecutive measurement of *L*-values vs. temperature yields an *L*(*T*) dependence, which reflects the molecular mobility and local rigidity of the matrix surrounding the spin probe. Since *L*(*T*) is measured based on relaxation times, it does not depend on the amount of detectable paramagnetic species. The theory of atomic displacements predicts that the *L*(*T*) grows linearly with temperature (Figure 1b) [35,41]. The slope of the *L*(*T*) characterizes the intensity of the librations. When glass softens and transforms into a liquid, the amplitude of the nitroxide motions grows drastically, manifesting a transition from librations to a free-diffusion regime. Thus, no other behaviors are generally expected of the *L*(*T*), unless some structural rearrangements occur; hence, the rising trend should not revert back down as *T* increases because the thermal energy, *kT,* driving the librations grows. Consequently, any deviations from the monotonic linear growth indicate structural changes in the glassy matrix surrounding the nitroxide. Typically, the anomalous suppression of the stochastic librations in ILs is found within ~150–200 K (region “Anomaly” in Figure 1b), wherein the exact range depends on the particular solvent. This nonlinear region of the *L*(*T*) is the most interesting finding and represents the structural rearrangements in IL glasses.

As demonstrated in our previous work [32], the alkyl chains govern the structural anomalies and the shape of the *L*(*T*) in ILs and IL-mimicking organic glasses. The drastic dependence of anomalies on the alkyl-chain length (i.e., the number of carbon atoms in the chain, *n*) has been evidenced. A series of [C_n_mim][BF_4_] Ils were studied extensively as a representative example, but the observed trends were found to be general (at least for imidazolium-based ILs). In brief, for short alkyl chains (*n* = 0, 1), no pronounced anomaly was detected. The chains with *n* = 2 induced the onset of librations at abnormally low temperatures of ~30 K, followed by a growth at *T*~30–90 K, which then reversed within the range, *T* = 90–150 K, displaying qualitatively similar anomalies to those observed for the intermediate-length (*n* = 3–10) group. In this group, the monotonous increase in librations began at ~80 K and continued up to a local maximum, *L*_max_, at *T*~(*T*_g_ − 60) K with the consequent suppression in librations within a range of (*T*_g_ − 60) K to *T*_g_ (the anomalous region). After this, a rapid growth occurs at temperatures higher than *T*_g_ due to the melting of the IL and its transition from small-angle librations to a diffusive rotation motion of the spin probe. For long alkyl chains (*n* > 10), almost no anomaly is observed. Remarkably, these intriguing structural anomalies were observed not only in ILs but also in other neutral glass-formers, for which its molecular shapes mimic the structure of IL cations, i.e., possessing sufficiently long alkyl chains [32]. For instance, this has been evidenced in a series of phthalates in the glassy state [42].

Thus far, general trends of nanostructuring and structural anomalies have not been studied in API-ILs. As discussed above, both API-ILs and nanostructuring/structural anomalies in ILs are of considerable interest. Therefore, for the first time, we investigated nanostructuring in a series of API-IL glasses using a previously developed EPR methodology. To make the current study of API-ILs more comprehensive, the cations with various alkyl-chain lengths (but within the range of the most pronounced anomaly, i.e., *n* = 2, 4, and 6) have been chosen for every selected anion. Below, we describe the results obtained, their interpretation, and conclusions on specifics of molecular nanostructuring in API-ILs.

## 2. Results and Discussion

**Scheme 1:**
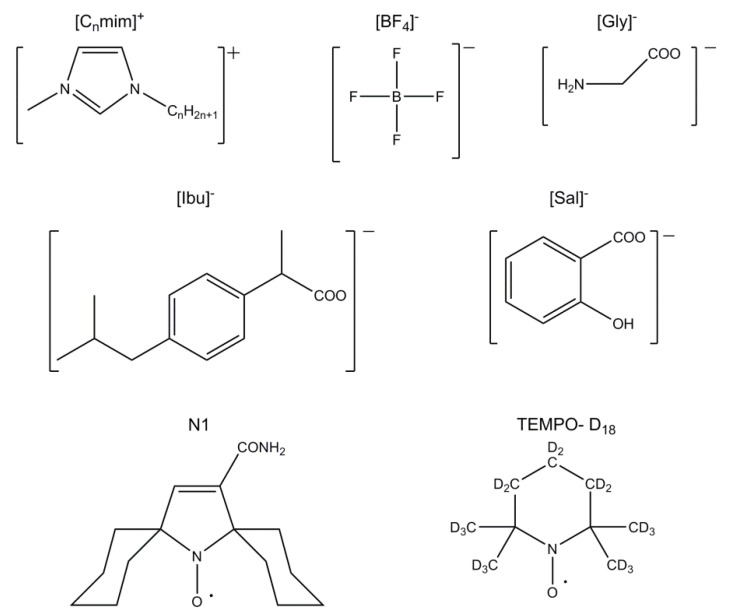
The chemical structures of the studied API-ILs and spin probes, N1 and TEMPO-D_18_.

In this study, we selected a set of API-ILs along with a few common ILs for comparison. All of them used [C_n_mim]^+^ cations (*n* = 2, 4, and 6), whereas [Ibu]^−^, [Gly]^−^, and [Sal]^−^ anions were used in API-ILs and [BF_4_]^−^ in regular ILs (Scheme 1).

**Figure 2:**
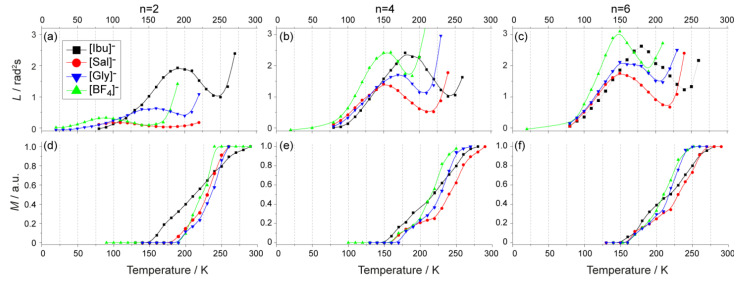
(**a**–**c**) The temperature dependence of the motional parameter, *L* ≈ 10^11^<α^2^>τ_c_, for nitroxide radical, N1; (**d**–**f**) the mobile fraction, *M,* for the nitroxide radical, TEMPO-D_18_, dissolved in selected API-ILs. The graphs are grouped by the same n in the cation alkyl chain. The *T*_g_ values are given in the text or the SI. Solid lines guide the eye. The error of each *M* value does not exceed ~30%.

Figure 2a–c show the *L*(*T*) dependencies obtained in three studied API-ILs, [C_n_mim][Ibu], [C_n_mim][Gly], and [C_n_mim][Sal], which were grouped by the length of the cationic alkyl chain, *n* = 2, 4, and 6. The results obtained for [C_n_mim][BF_4_] in our previous study [32] were added as a reference. Different types of characteristic *L*(*T*) behaviors were observed. For instance, Figure 2a shows that the *L*(*T*) for [C_2_mim][Ibu] dramatically differs from that of the [C_2_mim]-based ILs with other anions.

The *L*(*T*) is sensitive to the librational motion; it reflects a pronounced mobility of the probes located in glassy matrix, but it is insensitive to the much weaker mobility of the probes in crystalline surroundings. At the same time, the CW EPR is sensitive to the mobility of probes localized in any kind of matrix: from a liquid state, allowing the large-scale diffusive rotation of radicals, to a crystalline state, where radicals are completely immobilized. Therefore, the CW EPR allows distinguishing the two contributions into the observed signal vs. the temperature: the mobile *M*(*T*) and immobile (1 − *M*(*T*)) fractions [29,30]. Altogether, the information provided by *M*(*T*) and *L*(*T*) is complementary.

Following the procedure described in detail in our previous studies [29,30,31,32,42], we obtained the CW EPR spectra of the dissolved probe, TEMPO-D_18_, and simulated each spectrum as a superposition of the immobile and mobile fractions. Figure 2d–f show the temperature dependence of the mobile fraction, *M*(*T*), for selected API-ILs.

In all cases of ILs that demonstrate a structural anomaly, the onset of the mobile fraction, *M*(*T*), occurs close to a local maximum of *L*(*T*) (*L*_max_, Figure 1b). This implies the coexistence of two microenvironments in the anomaly region. However, in the case of [C_n_mim][Ibu], the rise in *M*(*T*) begins at ~30 K lower than the beginning of the anomaly in the *L*(*T*) curve; as a result, the fraction of mobile radicals reaches *M* = 0.8 at *T*_g_. Such behavior is quite different to that observed in other studied ILs: for those, the onset of *M*(*T*) strictly coincides with the beginning of the anomaly in the *L*(*T*), and the value *M*(*T*_g_)~0.2 is typically found.

Previously, we evidenced that the nitroxide probe is preferentially surrounded by non-polar alkyl moieties of imidazolium cations. Moreover, the length of the alkyl chain governs the structural anomaly’s evolution in the glassy IL [32], whereas the anion structure has a minor impact on the properties of the anomaly (for ILs with a fixed imidazolium cation); the anions mainly influence the *T*_g_ values [29]. However, the *L*(*T*) curves shown in Figure 2a–c demonstrate dramatically different results for various anions.

The series of ILs, [C_n_mim][Sal], demonstrates the lowest intensity of librations among the ILs with the four selected anions. The local maximum of *L*(*T*) for [C_2_mim][Sal] reaches the value *L*_max_ = 0.17, while the values *L*_max_ = 0.33, 0.63, and 1.9 were observed for [BF_4_]^−^, [Gly]^−^, and [Ibu]^−^, respectively. For [C_4_mim]^+^ and [C_6_mim]^+^ cations, this trend changes: ILs with [BF_4_]^−^ have the highest *L*_max_ value. However, the relative trend between [Sal]^−^-, [Gly]^−^-, and [Ibu]^−^-based ILs remains the same: the most pronounced librations are found for [Ibu]^−^, and they are intermediate for [Gly]^−^ and least intensive for [Sal]^−^.

We suppose that the obtained trends correlate with a general coordination ability of anions. [Sal]^−^ has the highest coordination ability due to the presence of two coordination sites: COO^−^ and OH^−^. The use of salicylate complexes for “cation hiding” is a well-known procedure in analytical chemistry [43]. Moreover, [Sal]^−^ tends to form hydrogen bonds with radical, N1. Strehmel et al. investigated how hydrogen bonding between the nitroxide radical and IL ions contributes to the mobility of a spin probe dissolved in heterogeneous IL media [44,45,46,47]. In general, the significantly reduced mobility of a spin probe in an IL with pronounced hydrogen bonding was demonstrated. Indeed, we cannot exclude the possibility of π-stacking between the salicylate anions or salicylate-imidazolium pairs, which will increase the rigidity of the IL matrix. This accords with the suppression in the librational motion of the probe in [C_n_mim][Sal]. In the case of [Ibu]^−^, the substantial non-polar fragment and the presence of only one coordination site make these anions less prone to coordinate with each other and with nitroxide, leading to a more fragile microstructure. The compact [Gly]^−^ anion with an “unshielded” COO^−^ coordination site corresponds to the intermediate case.

Thus, most unusual *L*(*T*) behaviors are observed for [C_n_mim][Ibu]. Considering the structures of studied API-ILs, the [Ibu] anion clearly stands out. The API-ILs, [C_n_mim][Ibu], present alkyl chains not only in cations but also as non-polar groups of anions. Indeed, this is the first example in our studies in which the alkyl fragments (*n* > 2) are present in both counter ions. As an additional factor, the size of the [Ibu]^−^ ion is comparable with that of the imidazolium cation. Therefore, it is plausible that the spin probe is located in the complex surroundings—and not simply in micelle-like cavities formed by alkyl chains of cations.

**Figure 3:**
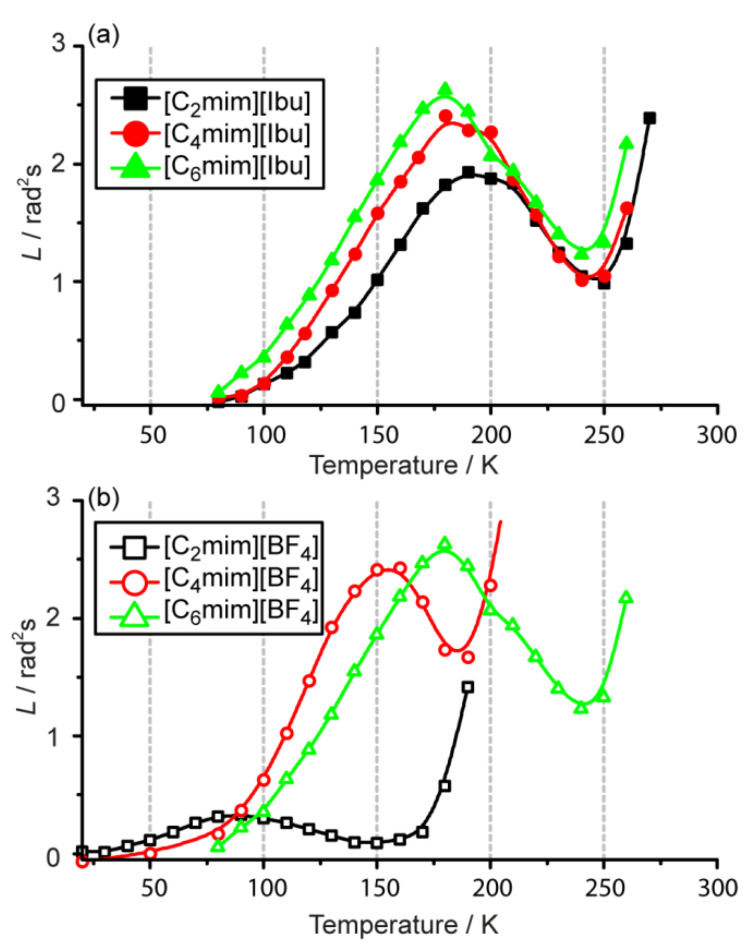
The temperature dependence of the motional parameter *L* ≈ 10^11^<α^2^>τ_c_ for the nitroxide radical N1 dissolved in (**a**) [C_n_mim][Ibu] and (**b**) [C_n_mim][BF_4_]. Solid lines guide the eye.

This assumption is indirectly supported by the example of [C_2_mim][Ibu], for which its *L*(*T*) shows a pronounced anomaly in the range of ~170–240 K (Figure 3a). Note that such an anomaly has never been observed for [C_2_mim]^+^-based ILs with various anions; therefore, we reasonably speculate that, in the case of [C_2_mim][Ibu], the spin probe is localized in the micelle-like cavity formed by [Ibu]^−^ anions. Interestingly, the absolute value of *L*(*T*) of [C_2_mim][Ibu] is close to that of [C_4_mim][BF_4_], which also supports the assumption that a non-polar fragment of an anion governs the anomaly in the [C_2_mim][Ibu] case. Furthermore, Figure 3 provides a clear comparison of the effect of the alkyl-chain length on the *L*(*T*) in the series, [C_n_mim][Ibu] and [C_n_mim][BF_4_], with *n* = 2, 4, and 6. It is evident that, in the case of [Ibu]^−^, the alkyl-chain length has a minor impact on the shape of the *L*(*T*) curve, while for [BF_4_]^−^, the chain length, n, drastically influences *L*(*T*). Thus, the [Ibu]^−^ ion strongly affects the general trends of “micelle-like” nanostructures formed around the dissolved radical.

It is worth noting that the *T*_g_ values of [C_n_mim][Ibu] are considerably lower than the local minimum of the *L*(*T*) curve, *L*_min_ (Table 1). In all other ILs that we studied, the *T*_g_ point actually terminates the anomaly region and manifests the transition from librations to a free diffusive motion of the probe; however, this is not the case for [C_n_mim][Ibu]. According to the CW EPR data (Figure 2d–f), the amount of “mobile” radicals grows very gradually with temperature such that the complete transition of a sample to its liquid state occurs close to room temperature (*M*(*T*) = 1); the entire glass-liquid transition of the radical-surrounding nanostructure takes more than 100 K [29].

**Figure 4:**
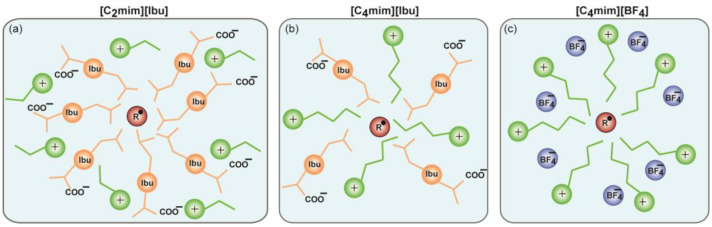
The images of the partly ordered hypothetical heterogeneous microstructures of representative ILs around the dissolved-radical probe. (**a**) Microstructure in case of IL with small alkyl moiety of cation: the micelle around the spin probe is formed by alkyl moieties of anions. (**b**) Microstructure in case of IL with alkyl moieties of comparable size in cations and anions: both kinds of ions form radical surrounding. (**c**) Microstructure in case of IL with no alkyl moiety in anion: the radical surrouinding is formed by aklyl chains of cations.

In this context, the mismatch between the *T*_g_ point and the *L*(*T*) local minimum, *L*_min_, for [C_n_mim][Ibu] also supports the assumption of a specific-radical surrounding, which is different from that of common imidazolium-based ILs. We propose that the non-polar moiety of the ibuprofene anion specifically shields the radical from the rest of the bulk IL with imidazolium cations. Figure 4 summarizes our assumptions on the peculiarities of nanostructuring in [C_n_mim][Ibu]. In the case of [C_2_mim][Ibu], the nitroxide is mainly surrounded by anions (Figure 4a); this makes the anomaly resemble that in [C_4_mim][BF_4_], for which its structure is a result of the micelle-like environment formed by the butyl chains of cations (Figure 4c). For the API-ILs, [C_4_mim][Ibu] and [C_6_mim][Ibu], the anomaly is slightly more intensive compared to that of [C_2_mim][Ibu]; therefore, we speculate that the micelle-like nanostructures in these API-ILs include “mixed-micelles” with both the involvement of cations and anions (Figure 4b).

In addition to the unusual behavior of [C_n_mim][Ibu], some intriguing peculiarities were found also in API-ILs with [Sal]^−^ and [Gly]^−^ anions. In particular, there is a notable curvature displayed by the *M*(*T*) dependence in the vicinity of the *T*_g_ point (Figure 2d,e). The curvature occurs at ~220 K for [C_2_mim][Sal], [C_4_mim][Sal], and [C_6_mim][Sal] and at ~210–220 K for the corresponding [Gly]^−^ API-ILs. The rapid rise in *M*(*T*) at higher temperatures may indicate a more efficient process of matrix softening that naturally occurs in the *T*_g_ vicinity and triggers the onset of diffusive motion. The same phenomenon is less pronounced for the [Ibu]^−^ API-ILs.

**Figure 5:**
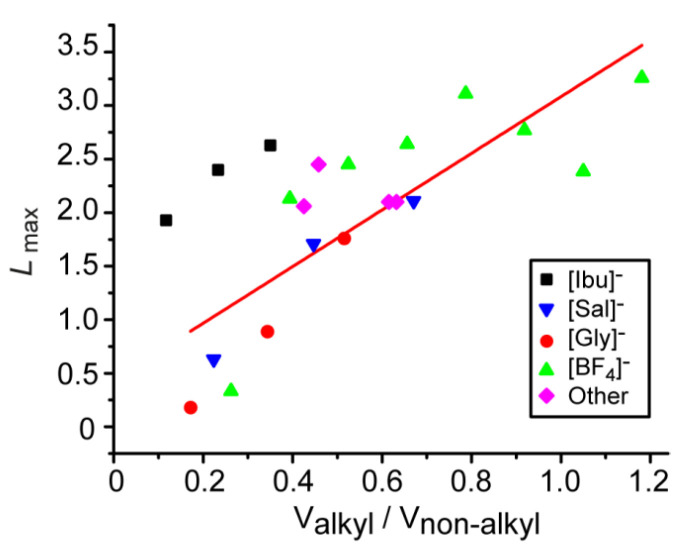
The maximum values of librations, *L*_max_, vs. the ratio of alkyl-chain volume to the volume of rest of the IL–ion couple. The red line shows the linear fit of the presented data, excluding results for [Ibu]^−^ API-ILs. Some data points are taken from previous research [28,29,30,32,33,42].

Apparently, [C_2_mim][Sal] has the most rigid matrix among all of the studied API-ILs; this stems from two independent factors. First, the high coordination ability of the anion stabilizes the matrix and makes it form close to a crystal-like structure. Second, the hydrophobic interaction between short non-polar alkyl chains of the cation is too weak to counteract the anion–anion interactions and form cation-based micelles around the spin probe. The maximum value of the libration parameter *L*_max_ is clearly connected with the overall rigidity of the glassy matrix. We found that there is a clear correlation between the relative volume of the alkyl chain and the *L*_max_. Figure 5 presents the data collected from this and previous studies of structural anomalies in glassy ILs. The vertical axis shows the *L*_max_ for the corresponding IL; the horizontal axis shows the ratio of alkyl-chain volume to the overall volume of the remaining part of the IL. The volumes of the anions [Gly]^−^, [Sal]^−^, and [Ibu]^−^ were estimated by the “atoms in molecules” (AIM) partitioning of electron-density distributions [59]. The geometries of the selected anions were optimized using DFT-B3LYP calculations in the Orca software [60] (coordinates of atoms obtained are given in the SI). Subsequently, we obtained the density distribution by AIM partitioning and analyzed it using the Multiwfn program [61]. The volumes of the [BF_4_]^−^, [PF_6_]^−^, [Cl]^−^, [Br]^−^, [CF_3_SO_3_]^−^, and [C_n_mim]^+^ ions were taken from the literature [59].

Figure 5 supports the general trends of the anomaly. The radical is surrounded by the micelle-like structure formed by alkyl chains of cations, and the larger the volume of alkyl chains, the less rigid the glass (for *n* < 10), leading to more pronounced librations (larger *L*_max_). In this regard, it is remarkable that [Ibu]^−^-based API-ILs are ‘outliers’ of the main trend, once again supporting the assumption of different nature of radical surrounding in this type of ILs.

## 3. Materials and Methods

All API-ILs were synthesized following the procedure described in the Supporting Information (SI). The spiro-cyclohexane-substituted nitroxide N1 and TEMPO-D_18_ were kindly provided by Dr. Igor Kirilyuk, NIOCh SB RAS. The radical was dissolved in the corresponding IL at a concentration of 1 mM, and the solution was placed in an EPR quartz tube with a 3.8 mm outer diameter. Then, it was evacuated at 10^−5^ mbar for 2 h to reduce the amount of remaining water and to eliminate the remaining oxygen; it was then exposed to 3–5 freeze–pump–thaw cycles and finally sealed off under a vacuum. The samples with TEMPO-D_18_ were prepared with the same method but excluded the heating step during pumping to avoid a radical reduction.

EPR measurements were taken using Bruker’s commercial Elexsys E580-spectrometer at the X-band. The spectrometer was equipped with an Oxford Instruments temperature-control system (4–300 K). The echo-detected EPR spectra and phase-memory times were recorded using the standard two-pulse echo sequence with the pulse lengths of approximately 100 ns for π and 50 ns for π/2. A monoexponential analysis of echo decay vs. 2τ yielded the corresponding *T*_2_ value in a certain magnetic field. The CW EPR spectra were acquired using Bruker’s X-band EMX spectrometer (9 GHz). In all experiments, the sample was first shock-frozen in liquid nitrogen and then transferred into the cryostat. In this way, we ensured that the sample was in the glassy state at the beginning of each experiment. The heating/cooling rates were ca. 1 K/min; the sample was equilibrated for at least 10 min prior to each EPR measurement. All spectral simulations were performed using EasySpin [62].

The details of the quantum-chemistry calculations are given in the SI.

## 4. Conclusions

In this work, we studied for the first time nanostructuring in a special class of ILs—API-ILs—which are of great interest and potential for various pharmaceutical applications. Since previous EPR methodology employing spin probes has been shown to be informative for studying nanostructures and structural anomalies in other ILs, we applied the same approach here to study a series of API-ILs: [C_n_mim][Ibu], [C_n_mim][Gly], and [C_n_mim][Sal] with *n* = 2, 4, and 6. Firstly, it has been demonstrated that structural anomalies associated with nonmonotonous molecular mobility vs. temperature are also displayed in API-ILs. At the same time, a new trend was found for [C_n_mim][Ibu]: This API-IL includes alkyl chains not only in cations but also in anions; therefore, mixed-anion/cation micelle-like environments form around the dissolved nitroxide probes. This further supports the key role that alkyl chains play in the occurrence of structural anomalies and also proposes a new tool to adjust and influence such behaviors by tuning the structure of both counter-ions. Although this knowledge is rather basic at the moment, we predict that, in future tuning applications of structural anomalies in bio-friendly ILs, e.g., API-ILs, this will open new opportunities for pharmaceutical and biomedical applications.

## Figures and Tables

**Table 1:** The glass-transition temperature of the selected ILs in [a]—this study (obtained from DSC data); [b]—literature data.

Ionic Liquid	*T*_g_^[a]^/K	*T*_g_^[b]^/K
[C_2_mim][Ibu]	234.15	
[C_4_mim][Ibu]	228.35	
[C_6_mim][Ibu]	228.45	
[C_2_mim][Sal]	209.25	
[C_4_mim][Sal]	202.85	
[C_6_mim][Sal]	219.55	
[C_2_mim][Gly]	205.35	
[C_4_mim][Gly]	201.15	
[C_6_mim][Gly]	196.35	
[C_2_mim][BF_4_]	167.9 *	186.15 [48] 180.15 [49] 184 [50] 183.75 [51] 181.15 [52] 178 [53]
[C_4_mim][BF_4_]	187.7 *	188.15 [52] 202 [54] 176.15 [55] 188.85 [56] 185.85 [57]
[C_6_mim][BF_4_]	188 *	190.75 [54] 187.55 [57] 194 [58]

(*) Ref. [32].

## Data Availability

Not applicable.

## Supplementary Materials

The following Supporting Information are available online: https://www.mdpi.com/article/10.3390/molecules27165117/s1. Details on Chemicals, Synthesis of API ionic liquids, The NMR spectral data of ionic liquids, DSC data, CW EPR data, and Details of quantum chemistry calculations. References [63,64,65] are cited in the supplementary materials

## Sample Availability

Samples of the compounds are not available from the authors.
