# Supplementary material for: Active Pharmaceutical Ingredient-Ionic Liquids (API-ILs): Nanostructure of the Glassy State Studied by Electron Paramagnetic Resonance Spectroscopy
Source: Molecules. 2022 Aug 11;27(16):5117. doi: 10.3390/molecules27165117 (PMC9415235; doi:10.3390/molecules27165117)
Supplement: Supplementary file 1 [file molecules-27-05117-s001.zip › molecules-1835502-supplementary.pdf]

SUPPORTING INFORMATION

Active Pharmaceutical Ingredient - Ionic Liquids (API-ILs): Nanostructure of glassy state studied by Electron Paramagnetic Resonance spectroscopy

Olga D. Bakulina [1,2], Mikhail Yu. Ivanov [1,2], Dmitriy V. Alimov [1,2], Sergey A. Prikhod'ko [3], Nicolay Yu. Adonin[3] and Matvey V. Fedin [1,2*]*

[1]International Tomography Center SB RAS, Institutskaya Street 3a, 630090 Novosibirsk, Russia

[2]Novosibirsk State University, Pirogova Street 2, 630090 Novosibirsk, Russia

[3]Boreskov Institute of Catalysis SB RAS, Lavrentiev Avenue 5, 630090 Novosibirsk, Russia

Table of Contents

Chemicals 2
Synthesis of ibuprofen and salicylic acid potassium salts 2
Synthesis of ionic liquids 2
The NMR spectral data of ionic liquids 3
DSC measurements 4
Quantum chemical calculations 6
Continuous wave EPR 8
References 13

Chemicals

1-Chlorobutane, 1-chlorohexane, N-methyl imidazole, 1-ethyl-3-methyl imidazolium chloride, glycine, salicylic acid, ibuprofen, Dowex 1x8 ion exchange resin (100-200 mesh) and all standard chemicals were obtained from commercial sources.

The ionic liquids [C_4mim]Cl and [C_6mim]Cl were obtained by analogy with the literature method [S1] from 1-methyl imidazole and chlorobutane or chlorohexane.

Synthesis of ibuprofen and salicylic acid potassium salts

Ibuprofen potassium

8.251 g (40 mmol) of ibuprofen was dissolved in 50 ml of hot ethanol and then 50 ml of water was added. A solution of potassium hydroxide (2.244 g (40 mmol) in 20 ml of water) was added dropwise to the resulting suspension. The resulting mixture was stirred for 30 minutes at 50°C. pH of the resulting mixture was about 8. Water was evaporated in vacuum. The product was recrystallized from acetonitrile. 8.1 g (83%) of potassium ibuprofene was obtained.

Potassium salicylate

15.0 g (108.5 mmol) of salicylic acid was suspended in 100 ml of hot water (~50°C). A solution of potassium hydroxide (6.087 g (108.5 mmol) in 20 ml of water) was added dropwise to the resulting suspension. The resulting mixture was stirred for 30 minutes at 50°C. pH of the resulting mixture was about 7. Water was evaporated, and resulting salt was dried in high vacuum at 80°C. The yield of potassium salicylate was almost quantitative.

Synthesis of ionic liquids

Ibu- and Sal- ionic liquids (general procedure)

A 100 ml round-bottomed flask fitted with magnetic stirrer bar and reflux condenser was charged by 15 mmol of corresponding ionic liquid ([C_2mim]Cl, [C_4mim]Cl or [C_6mim]Cl), 22 mmol of corresponding salt (IbuK or SalK) and 70 ml of acetonitrile. The mixture was stirred at 60°C for 6 hours. The precipitate was filtered off, and the solvent was evaporated in vacuo. The residue was dissolved in 30 ml of dichlorometane. The precipitate was filtered off, and the solvent was evaporated in vacuo. The product was dried in high vacuum (10^{-3} bar) at 60°C for 6 hours. Yield of products are 75-85 %. The reaction of sample of ionic liquid with $AgNO_3$ was negative*, that demonstrates the absence of Cl– ions in the ionic liquid media.

* Since silver salts with ibuprofen and salicylic acid are insoluble in water, concentrated nitric acid was added to the sample to eliminate the influence of anions. HNO_3 converts organic anions into acids, but does not affect the determination of chloride ions in solution.

Gly- ionic liquids (general procedure, by analogy with [S2])

20 ml of Dowex 1x8 ion exchange resin (Cl- form, 1.2 mEq/ml, 23 mmol-Eq) were loaded onto the column. To convert ion exchange resin to the OH form, a 1M NaOH solution (250 ml, 250 mmol) was passed through the column, after which water was passed through the column until pH 7 was reached in the wash.

After that, a solution of 11.5 mmol of the corresponding ionic liquid ([C_2mim]Cl, [C_4mim]Cl or [C_6mim]Cl) in 100 ml of water was passed through the column at a rate of 0.1-0.2 ml/sec. At the end of the passage, the column was washed with another 100 ml of water. The combined solution was added

dropwise to a solution of 1.04 g (13.8 mmol) of glycine in 100 ml of ice water. The mixture was stirred at room temperature for 12 hours, after which the water was distilled off on a rotary evaporator at a temperature not exceeding 45°C. A mixture of 45 ml of acetonitrile and 5 ml of methanol was added to the residue. The resulting suspension was stirred for 30 minutes, the precipitate was filtered off, the solvents were distilled off in a vacuum. The product was dried in high vacuum (10^{-3} bar) at 60°C for 16 hours. Yield of products are 85-90 %. The reaction of sample of ionic liquid with $AgNO_3$ was negative*, that demonstrates the absence of Cl– ions in the ionic liquid media.

* Since silver salt with glycine is insoluble in water, concentrated nitric acid was added to the sample to eliminate the influence of anion. HNO_3 converts glycine into nitric acid salt, but does not affect the determination of chloride ions in solution.

The NMR spectral data of ionic liquids

The 1H NMR spectra were recorded on a Bruker AVANCE 300 spectrometer at 300.13 MHz, the chemical shifts are referenced to TMS.

1-Ethyl-3-methylimidazolium ibuprofen ([C_2mim]Ibu)

δ (CD_3CN) 10.24 (s, 1H, *H*-2); 6.92-7.26 (m, 6H, *H*-aromatic (Ibu), *H-4,5*); 4.05 (q, 2H, $^3J_{HH}$ 7.3 Hz, N-CH_2-CH_3); 3.68 (s, 3H, N-CH_3); 3.54 (q, 1H, , $^3J_{HH}$ 7.0 Hz, CH_3-C*H*-COO (Ibu)); 2.34 (d, 2H, , $^3J_{HH}$ 7.1 Hz, CH_2-CH-$(CH_3)_2$ (Ibu)); 1.75 (st, 1H, $^3J_{HH}$ 6.7 Hz, CH_2-C*H*-$(CH_3)_2$ (Ibu)); 1.31-1.41 (m, 6H, CH_3-CH-COO (Ibu), N-CH_2-CH_3); 0.83 (d, 6H, $^3J_{HH}$ 6.5 Hz, CH_2-CH-(CH_3)$_2$ (Ibu)).

1-Butyl-3-methylimidazolium ibuprofen ([C_4mim]Ibu)

δ (CD_3CN) 9.91 (s, 1H, *H*-2); 6.91-7.24 (m, 6H, *H*-aromatic (Ibu), *H-4,5*); 4.00 (t, 2H, $^3J_{HH}$ 7.3 Hz, N-CH_2); 3.67 (s, 3H, N-CH_3); 3.49 (q, 1H, , $^3J_{HH}$ 7.0 Hz, CH_3-C*H*-COO (Ibu)); 2.32 (d, 2H, , $^3J_{HH}$ 7.6 Hz, CH_2-CH-$(CH_3)_2$ (Ibu)); 1.60-1.80 (m, 3H, CH_2-C*H*-$(CH_3)_2$ (Ibu) 2-CH_2); 1.32 (d, 3H, $^3J_{HH}$ 7.2 Hz, CH_3-CH-COO (Ibu)); 1.25 (m, 2H, 3-CH_2); 0.79-0.87 (m, 9H, CH_2-CH-(CH_3)$_2$ (Ibu), 4-CH_3).

1-Hexyl-3-methylimidazolium ibuprofen ([C_6mim]Ibu)

δ (CD_3CN) 10.41 (s, 1H, *H*-2); 6.98-7.28 (m, 6H, *H*-aromatic (Ibu), *H-4,5*); 4.10 (t, 2H, $^3J_{HH}$ 7.4 Hz, N-CH_2); 3.81 (s, 3H, N-CH_3); 3.60 (q, 1H, , $^3J_{HH}$ 7.0 Hz, CH_3-C*H*-COO (Ibu)); 2.39 (d, 2H, , $^3J_{HH}$ 7.2 Hz, CH_2-CH-$(CH_3)_2$ (Ibu)); 1.79 (st, 1H, $^3J_{HH}$ 6.7 Hz CH_2-C*H*-$(CH_3)_2$ (Ibu)) 1.40 (d, 2H, , $^3J_{HH}$ 6.8 Hz, CH_3-CH-COO (Ibu)); 1.39-1.51 (m, 2H, 2-CH_2); 1.27 (m, 6H, 3-5-CH_2); 0.86-0.92 (m, 9H, CH_2-CH-(CH_3)$_2$ (Ibu), 6-CH_3).

1-Ethyl-3-methylimidazolium salicylate ([C_2mim]Sal)

δ ($CDCl_3$) 10.00 (s, 1H, *H*-2); 7.79 (dd, 1H, , $^3J_{HH}$ 7.7 Hz, $^5J_{HH}$ 1.6 Hz, *H*-aromatic (Sal)); 7.25-7.37 (m, 3H, *H*-aromatic (Sal), *H-4,5*); 6.76-6.86 (m, 2H, *H*-aromatic (Sal));4.24 (q, 2H, $^3J_{HH}$ 7.4 Hz, N-CH_2-CH_3); 3.96 (s, 3H, N-CH_3); 1.51 (t, 3H, $^3J_{HH}$ 7.4 Hz, N-CH_2-CH_3).

1-Butyl-3-methylimidazolium salicylate ([C_4mim]Sal)

δ ($CDCl_3$) 10.05 (s, 1H, *H*-2); 7.82 (dd, 1H, , $^3J_{HH}$ 7.7 Hz, $^5J_{HH}$ 1.6 Hz, *H*-aromatic (Sal)); 7.08-7.20 (m, 3H, *H*-aromatic (Sal), *H-4,5*); 6.65-6.77 (m, 2H, *H*-aromatic (Sal));4.08 (t, 2H, $^3J_{HH}$ 7.4 Hz, N-CH_2); 3.90 (s, 3H, N-CH_3); 1.70 (tt, 2H, $^3J_{HH}$ 7.5 Hz, $^3J_{HH}$ 7.6 Hz, 2-CH_2); 1.22 (qt, 2H, $^3J_{HH}$ 7.4 Hz, $^3J_{HH}$ 7.5 Hz, 3-CH_2); 0.83 (t, 3H, $^3J_{HH}$ 7.3 Hz, 4-CH_3).

1-Hexyl-3-methylimidazolium salicylate ([C$_6$mim]Sal)

δ ($CDCl_3$) 9.75 (s, 1H, *H*-2); 7.76 (dd, 1H, , $^3J_{HH}$ 7.7 Hz, $^5J_{HH}$ 1.7 Hz, *H*-aromatic (Sal)); 7.17-7.34 (m, 3H, *H*-aromatic (Sal), *H-4,5*); 6.69-6.81 (m, 2H, *H*-aromatic (Sal));4.07 (t, 2H, $^3J_{HH}$ 7.5 Hz, N-CH_2); 3.91 (s, 3H, N-CH_3); 1.73 (m, 2H, 2-CH_2); 1.21 (m, 6H, 3-5-CH_2); 0.84 (t, 3H, $^3J_{HH}$ 6.7 Hz, 6-CH_3).

1-Ethyl-3-methylimidazolium glicynate ([C$_2$mim]Gly)

δ (dmso-d6) 9.92 (s, 1H, *H*-2); 7.85 (d, 2H, , $^3J_{HH}$ 13.4 Hz, *H-4,5*); 4.21 (q, 2H, $^3J_{HH}$ 7.4 Hz, N-CH_2-CH_3); 3.89 (s, 3H, N-CH_3); 2.71 (s, 2H, CH_2 *(Gly))*; 1.53 (t, 3H, $^3J_{HH}$ 7.5 Hz, N-CH_2-CH_3).

1-Butyl-3-methylimidazolium glicynate ([C$_4$mim]Gly)

δ (dmso-d6) 9.89 (s, 1H, *H*-2); 7.79 (d, 2H, , $^3J_{HH}$ 19.5 Hz, *H-4,5*); 4.16 (t, 2H, $^3J_{HH}$ 7.2 Hz, N-CH_2-CH_3); 3.84 (s, 3H, N-CH_3); 2.67 (s, 2H, CH_2 *(Gly))*; 1.73 (tt, 2H, $^3J_{HH}$ 7.4 Hz, $^3J_{HH}$ 7.4 Hz, 2-CH_2); 1.19 (qt, 2H, $^3J_{HH}$ 7.3 Hz, $^3J_{HH}$ 7.5 Hz, 3-CH_2); 0.84 (t, 3H, $^3J_{HH}$ 7.5 Hz, 4-CH_3).

1-Hexyl-3-methylimidazolium glicynate ([C$_6$mim]Gly)

δ (dmso-d6) 10.46 (s, 1H, *H*-2); 7.64 (d, 2H, , $^3J_{HH}$ 3.8 Hz, *H-4,5*); 4.18 (t, 2H, $^3J_{HH}$ 7.2 Hz, N-CH_2-CH_3); 3.87 (s, 3H, N-CH_3); 2.86 (s, 2H, CH_2 *(Gly))*; 1.76 (tt, 2H, $^3J_{HH}$ 7.1 Hz, $^3J_{HH}$ 7.1 Hz, 2-CH_2); 1.21 (m, 6H, 3-5-CH_2); 0.80 (t, 3H, $^3J_{HH}$ 6.9 Hz, 6-CH_3).

DSC measurements

DSC measurements were performed using DSC 204 F1 (NETZSCH) equipment at argon atmosphere with 30 ml/min flow rate. Samples were placed in the closed and pressed alumina melting pot with 25 μl volume. Setup calibration (on temperature and thermal capacity) was done using indium (99,999%) and zinc (99.8+%) purchased from Sigma Aldrich.

The samples were quickly cooled to -125 °C, after holding at the lowest *T* for 5 minutes were heated to the room temperature (25° C). Cooling and heating rate is always 10 ° C/min. The weight of the samples did not change.

Results are presented at Table S1 and Figures S1-S4.

Table S1. Glass transition temperatures of studied samples determined by DSC.

№	Sample	T_g onset, °C	Inflection[1], °C	ΔC_p, J/(g·K)
1	[C$_2$mim][Ibu]	-39.0	-34.9	0.297
2	[C$_4$mim][Ibu]	-44.8	-41.7	0.366
3	[C$_6$mim][Ibu]	-44.7	-36.7	0.370
4	[C$_2$mim][Gly]	-67.8	-63.5-	0.505
5	[C$_4$mim][Gly]	-72.0	-67.5	0.509
6	[C$_6$mim][Gly]	-76.8	-74.2	0.539
7	[C$_2$mim][Sal]	-63.9	-61.5	0.461
8	[C$_4$mim][Sal]	-70.3	-67.7	0.592
9	[C$_6$mim][Sal]	-53.6	-46.4	0.214

1 – The turning point between the begging and the end of glass transition

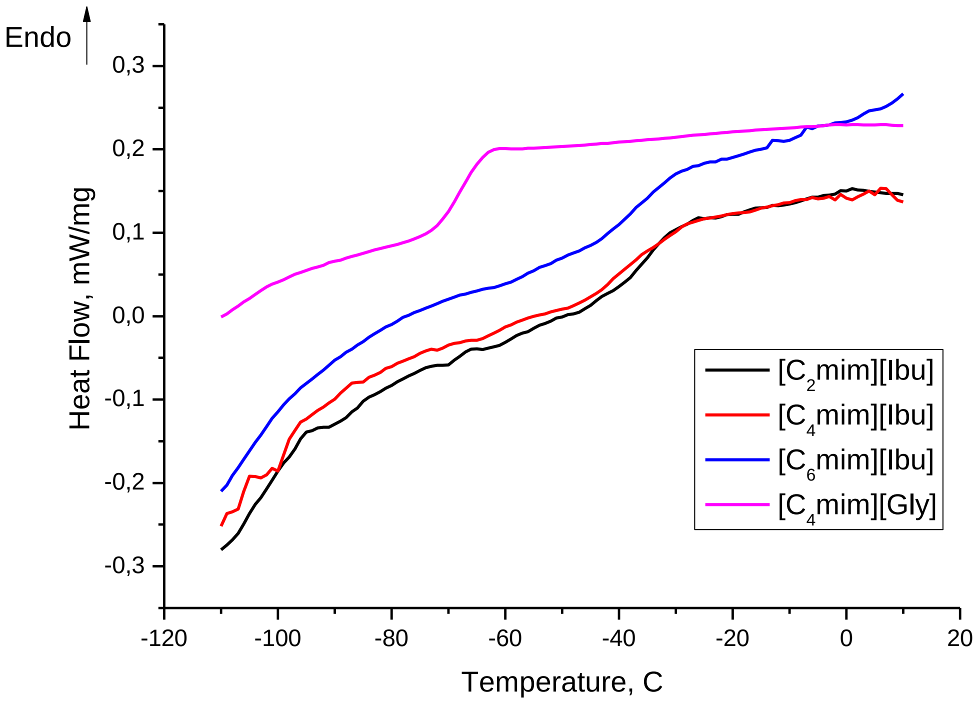

Figure S1. Overlay of DSC endotherms for [C_2mim][Ibu], [C_4mim][Ibu], [C_6mim][Ibu] and [C_4mim][Gly].

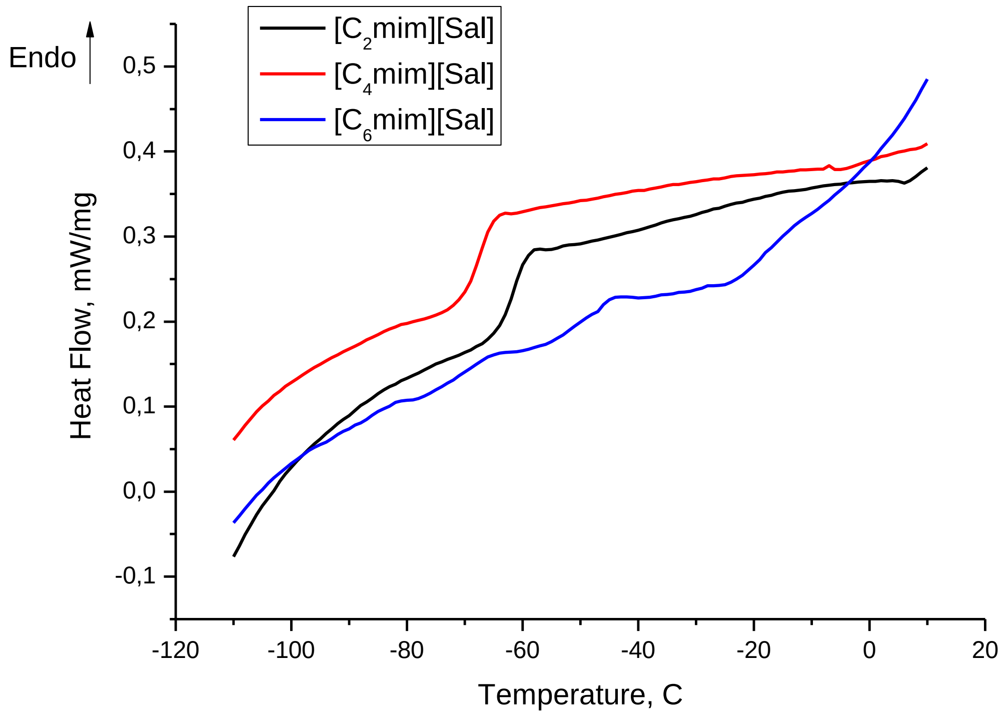

Figure S2. Overlay of DSC endotherms for [C_2mim][Sal], [C_4mim][Sal], [C_6mim][Sal].

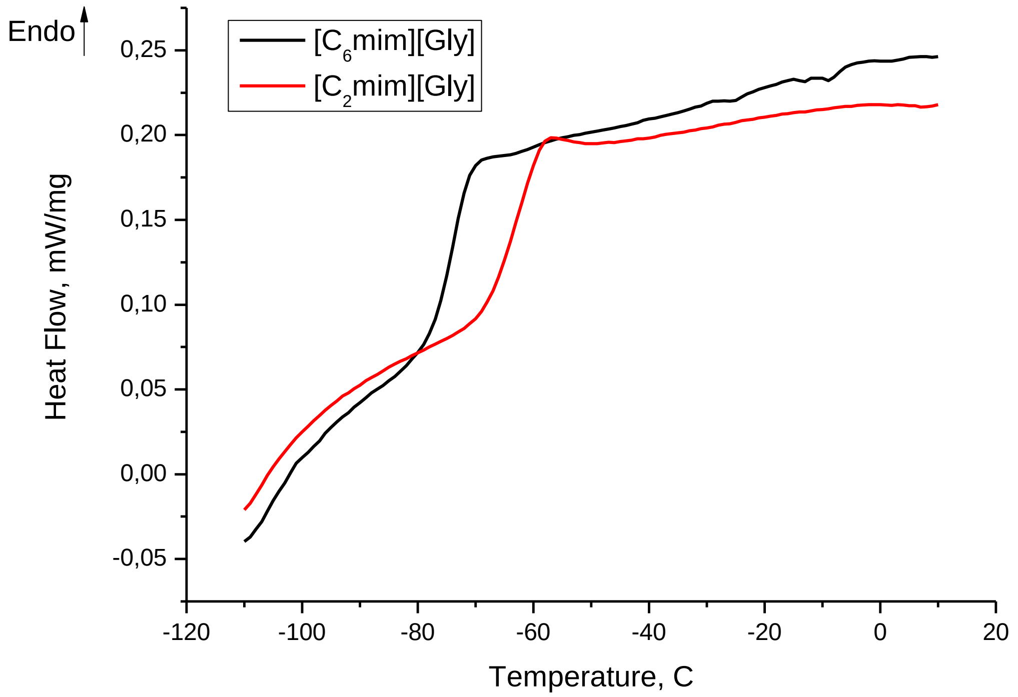

Figure S3. Overlay of DSC endotherms for [C_2mim][Gly], [C_6mim][Gly].

Quantum chemical calculations

Quantum chemical calculations were performed using the Orca software [S3]. For geometries optimization of selected anions DFT-B3LYP method was applied. The results of optimization are shown in Tables S2-S4.

Table S2. List of coordinates obtained during geometry optimization for $[Gly]^-$.

Atom	x, Å	y, Å	z, Å
N	-2.340878	-1.178282	0.078512
C	-2.088688	0.271180	0.209000
C	-0.614310	0.712025	-0.076974
O	-0.436134	1.944255	-0.182148
O	0.225979	-0.219510	-0.137487
H	-2.523706	-1.368204	-0.903545
H	-1.411601	-1.582875	0.221492
H	-2.319707	0.585702	1.234299
H	-2.759654	0.835808	-0.442950

Table S3. List of coordinates obtained during geometry optimization for $[Sal]^-$.

Atom	x, Å	y, Å	z, Å
C	0.781905	0.489218	-0.259526
C	1.329732	-0.743858	0.082024
C	0.478935	-1.775815	0.480095
C	-0.893182	-1.579221	0.535439
C	-1.447760	-0.334910	0.191075
C	-0.589224	0.717427	-0.214869
C	-1.171218	2.074215	-0.594259

O	-0.410553	2.980505	-0.946765
O	-2.453396	2.146928	-0.506749
O	-2.765341	-0.147044	0.245297
H	1.407510	1.316248	-0.574674
H	2.401845	-0.901841	0.038974
H	0.890254	-2.743736	0.750466
H	-1.563861	-2.373475	0.843338
H	-2.850946	0.875858	-0.070168

Table S4. List of coordinates obtained during geometry optimization for $[Ibu]^-$.

Atom	x, Å	y, Å	z, Å
C	2.290135	1.041633	-0.542385
C	2.657239	-0.297375	-0.700416
C	1.635550	-1.247513	-0.675211
C	0.310008	-0.873448	-0.481490
C	-0.058674	0.464590	-0.306947
C	0.967000	1.418218	-0.348738
C	-1.498066	0.856873	-0.104160
C	-1.724105	1.525799	1.259717
C	-2.011033	1.818348	-1.267081
O	-2.824008	1.301756	-2.056957
O	-1.544169	2.978760	-1.241090
C	4.098729	-0.710051	-0.885015
C	4.759766	-1.355859	0.353517
C	6.142152	-1.912975	0.000714
C	4.845752	-0.385036	1.532389
H	3.058240	1.808935	-0.582486
H	1.876315	-2.297469	-0.819830
H	-0.463316	-1.634408	-0.476540
H	0.694434	2.463584	-0.277986
H	-2.105694	-0.048404	-0.162288
H	-2.789778	1.719006	1.411454
H	-1.366716	0.898539	2.084764
H	-1.209073	2.485639	1.294184
H	4.169246	-1.423436	-1.714368
H	4.693003	0.163024	-1.179918
H	4.125294	-2.196522	0.658793
H	6.604297	-2.406336	0.860702
H	6.081278	-2.641713	-0.812492
H	6.815816	-1.111856	-0.320789
H	5.266910	-0.874691	2.415306
H	3.862715	0.005529	1.796841
H	5.488550	0.467059	1.287898

Continuous wave EPR

Figure S4 shows the CW EPR spectra of spin probe TEMPO-D_{18} dissolved in three API-ILs (indicated). The computer simulations were performed using EasySpin [S4]. Tables S5-S13 list the spectroscopic parameters used in CW EPR spectra of these and all other API-ILs.

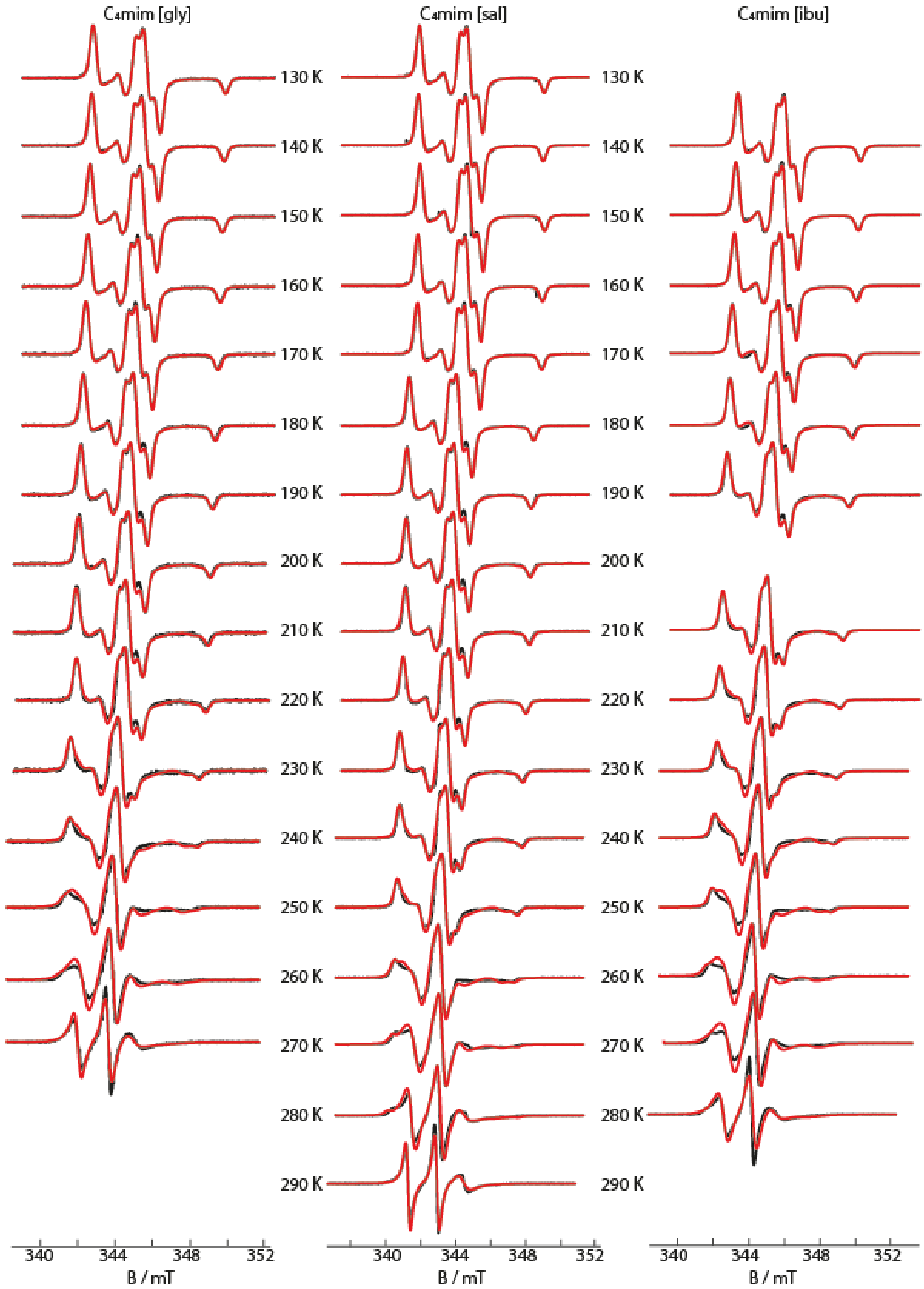

Figure S4. CW EPR spectra of TEMPO-D_{18} dissolved in three API-ILs (indicated) vs. temperature. Black lines show experimental data, red lines - the simulation results.

Table S5. List of parameters used in simulation of CW EPR spectra of TEMPO-D_{18} in [C_2mim][Gly].

		Mobile fraction			Immobile fraction		
T / K	M	τ_c / ns	g-tensor	A-tensor / MHz	g-tensor	A-tensor / MHz	Astrain / MHz
140	0	-	[2.0109 2.0073 2.0033]	[18.3 19.8 97.4]	[2.0109 2.0073 2.0033]	[18.3 19.8 97.4]	[3.3 0 6.0]
150	0	-					
160	0	-					
170	0	-					
180	0	-					
190	0	-					
200	0.12	5.9					
210	0.17	9.7					
220	0.24	11.3		[18.3 19.8 96.8]		[18.3 19.8 96.8]	
230	0.41	10.6					
240	0.58	10.2					
250	0.86	11.9		[18.3 19.8 97.8]		[18.3 19.8 97.8]	
260	1	4.13		[18.3 19.8 108.4]		[18.3 19.8 108.4]	

Table S6. List of parameters used in simulation of CW EPR spectra of TEMPO-D_{18} in [C_4mim][Gly].

		Mobile fraction			Immobile fraction		
T / K	M	τ_c / ns	g-tensor	A-tensor / MHz	g-tensor	A-tensor / MHz	Astrain / MHz
130	0	-	[2.0108 2.0071 2.0031]	[19.2 19.6 98.8]	[2.0108 2.0071 2.0031]	[19.2 19.6 98.8]	[4.5 2.6 5.6]
140	0	-					
150	0	-					
160	0	-					
170	0	-					
180	0.12	5.7					
190	0.17	8.0					
200	0.23	10.1					
210	0.28	8.9					
220	0.37	8.5		[19.2 19.6 96.6]		[19.2 19.6 96.6]	
230	0.54	8.2					
240	0.75	7.8					
250	0.94	6.3		[19.2 19.6 100.0]		[19.2 19.6 100.0]	
260	0.97	4.6		[19.2 19.6 101.1]		[19.2 19.6 101.1]	
270	1	2.9		[19.2 19.6 101.4]		[19.2 19.6 101.4]	

Table S7. List of parameters used in simulation of CW EPR spectra of TEMPO-D_{18} in [C_6mim][Gly].

	Mobile fraction	Immobile fraction

T / K	M	τ_c / ns	g-tensor	A-tensor / MHz	g-tensor	A-tensor / MHz	Astrain / MHz
130	0	-	[2.0108 2.0072 2.0032]	[18.3 20.0 96.5]	[2.0108 2.0072 2.0032]	[18.3 20.0 96.5]	[5.0 0 6.9]
140	0	-					
150	0	-					
160	0	-					
170	0.09	7.3					
180	0.15	8.2					
190	0.20	7.6					
200	0.30	7.6					
210	0.33	7.5		[18.3 20.0 95.1]		[18.3 20.0 95.1]	
220	0.57	7.6					
230	0.76	8.7					
240	0.97	7.2		[18.3 20.0 99.7]		[18.3 20.0 99.7]	
250	0.99	4.7		[18.3 20.0 105.1]		[18.3 20.0 105.1]	
260	1	4.2		[18.3 20.0 101.5]		[18.3 20.0 101.5]	
270	1	3.0		[18.3 20.0 100.7]		[18.3 20.0 100.7]	

Table S8. List of parameters used in simulation of CW EPR spectra of TEMPO-D_{18} in [C_2mim][Sal].

		Mobile fraction			Immobile fraction		
T / K	M	τ_c / ns	g-tensor	A-tensor / MHz	g-tensor	A-tensor / MHz	Astrain / MHz
140	0	-	[2.0110 2.0076 2.0036]	[19.5 19.4 101.1]	[2.0110 2.0076 2.0036]	[19.5 19.4 101.1]	[3.9 7.2 5.8]
150	0	-					
160	0	-					
170	0	-					
180	0	-					
190	0.07	6.0					
200	0.15	9.0					
210	0.24	10.4					
220	0.31	8.2		[19.5 19.4 99.2]		[19.5 19.4 99.2]	
230	0.50	8.0					
240	0.72	7.7					
250	0.91	5.5					
260	1	2.9		[19.5 19.4 101.4]		[19.5 19.4 101.4]	

Table S9. List of parameters used in simulation of CW EPR spectra of TEMPO-D_{18} in [C_4mim][Sal].

		Mobile fraction			Immobile fraction		
T / K	M	τ_c / ns	g-tensor	A-tensor / MHz	g-tensor	A-tensor / MHz	Astrain / MHz

130	0	-	[2.0108 2.0072 2.0032]	[19.2 19.5 99.0]	[2.0108 2.0072 2.0032]	[19.2 19.5 99.0]	[6.0 0 5.7]
140	0	-					
150	0	-					
160	0	-					
170	0.08	11.1					
180	0.14	9.3					
190	0.18	8.5					
200	0.21	9.5					
210	0.24	8.5					
220	0.25	7.3		[19.2 19.5 97.9]		[19.2 19.5 97.9]	
230	0.36	7.5					
240	0.51	8.2					
250	0.62	7.9		[19.2 19.5 96.0]		[19.2 19.5 96.0]	
260	0.81	6.9					
270	0.90	4.6		[19.2 19.5 99.7]		[19.2 19.5 99.7]	
280	0.94	3.1		[19.2 19.5 103.8]		[19.2 19.5 103.8]	
290	1	2.0		[19.2 19.5 100.9]		[19.2 19.5 100.9]	

Table S10. List of parameters used in simulation of CW EPR spectra of TEMPO-D_{18} in [C_6mim][Sal].

		Mobile fraction			Immobile fraction		
T / K	M	τ_c / ns	g-tensor	A-tensor / MHz	g-tensor	A-tensor / MHz	Astrain / MHz
130	0	-	[2.0110 2.0074 2.0037]	[19.2 19.5 98.9]	[2.0110 2.0074 2.0037]	[19.2 19.5 98.9]	[5.7 3.4 5.5]
140	0	-					
150	0	-					
160	0	-					
170	0.12	6.5					
180	0.16	8.4					
190	0.20	7.9					
200	0.25	8.1					
210	0.32	8.5					
220	0.35	7.3		[19.2 19.5 97.0]		[19.2 19.5 97.0]	
230	0.50	8.0					
240	0.64	8.0		[19.2 19.5 95.9]		[19.2 19.5 95.9]	
250	0.73	7.0					
260	0.92	5.8		[19.2 19.5 99.3]		[19.2 19.5 99.3]	
270	1	4.0		[19.2 19.5 102.7]		[19.2 19.5 102.7]	
280	1	2.8		[19.2 19.5 100.6]		[19.2 19.5 100.6]	

Table S11. List of parameters used in simulation of CW EPR spectra of TEMPO-D_{18} in [C_2mim][Ibu].

		Mobile fraction			Immobile fraction		
T / K	M	τ_c / ns	g-tensor	A-tensor / MHz	g-tensor	A-tensor / MHz	Astrain / MHz
130	0	-	[2.0107 2.0070 2.0029]	[19.2 19.9 96.8]	[2.0107 2.0070 2.0029]	[19.2 19.9 96.8]	[5.8 1.3 5.9]
140	0	-					
150	0	-					
160	0.08	7.4					
170	0.19	7.5					
180	0.26	8.7					
190	0.32	9.1					
200	0.40	8.5					
210	0.48	8.4					
220	0.56	8.5					
230	0.65	8.4					
240	0.74	8.3					
250	0.79	6.9		[19.2 19.9 96.0]		[19.2 19.9 96.0]	
260	0.89	6.5		[19.2 19.9 96.9]		[19.2 19.9 96.9]	
270	0.94	5.5		[19.2 19.9 98.0]		[19.2 19.9 98.0]	
280	0.97	4.4		[19.2 19.9 100.0]		[19.2 19.9 100.0]	
290	1	3.5		[19.2 19.9 98.3]		[19.2 19.9 98.3]	

Table S12. List of parameters used in simulation of CW EPR spectra of TEMPO-D_{18} in [C_4mim][Ibu].

		Mobile fraction			Immobile fraction		
T / K	M	τ_c / ns	g-tensor	A-tensor / MHz	g-tensor	A-tensor / MHz	Astrain / MHz
140	0	-	[2.0107 2.0070 2.0030]	[19.2 19.7 96.8]	[2.0107 2.0070 2.0030]	[19.2 19.7 96.8]	[6.9 0.1 6.3]
150	0	-					
160	0.07	4.4					
170	0.16	5.1					
180	0.21	5.3					
190	0.31	7.8					
210	0.42	7.9		[19.2 19.7 95.2]		[19.2 19.7 95.2]	
220	0.52	7.7					
230	0.62	8.2					
240	0.71	7.4					
250	0.80	6.6					
260	0.92	5.7		[19.2 19.7 98.3]		[19.2 19.7 98.3]	
270	0.98	4.8		[19.2 19.7		[19.2 19.7	

				101.4]		101.4]	
280	1	3.4		[19.2 19.7 104.2]		[19.2 19.7 104.2]	

Table S13. List of parameters used in simulation of CW EPR spectra of TEMPO-D_{18} in [C_6mim][Ibu].

			Mobile fraction		Immobile fraction		
T / K	M	τ_c / ns	g-tensor	A-tensor / MHz	g-tensor	A-tensor / MHz	Astrain / MHz
130	0	-	[2.0106 2.0070 2.0029]	[19.3 19.9 96.8]	[2.0106 2.0070 2.0029]	[19.3 19.9 96.8]	
140	0	-					
150	0	-					
160	0.06	6.4					
170	0.09	5.1					
180	0.23	5.6					
190	0.32	7.7					
200	0.39	8.2					
210	0.46	7.8		[19.3 19.9 95.2]		[19.3 19.9 95.2]	
220	0.50	7.7					
230	0.60	7.5					
240	0.71	7.4					
250	0.80	6.8					
260	0.92	6.0		[19.3 19.9 98.0]		[19.3 19.9 98.0]	
270	0.96	5.4		[19.3 19.9 99.3]		[19.3 19.9 99.3]	
280	1	3.3		[19.3 19.9 98.4]		[19.3 19.9 98.4]	

References

S1. Dupont, J.; Consorti, C.S.; Suarez, P. a. Z.; De Souza, R.F. PREPARATION OF 1-BUTYL-3-METHYL IMIDAZOLIUM-BASED ROOM TEMPERATURE IONIC LIQUIDS. *Org. Synth.* **2002**, *79*, 236, doi:10.15227/orgsyn.079.0236.

S2. Fukumoto, K.; Yoshizawa, M.; Ohno, H. Room temperature ionic liquids from 20 natural amino acids. *J. Am. Chem. Soc.* **2005**, *127*, 2398–2399, doi:10.1021/ja043451i.

S3. Neese, F. The ORCA program system. *Wiley Interdiscip. Rev. Comput. Mol. Sci.* **2012**, *2*, 73–78, doi:10.1002/wcms.81.

S4. Stoll, S.; Schweiger, A. EasySpin, a comprehensive software package for spectral simulation and analysis in EPR. *J. Magn. Reson.* **2006**, *178*, 42–55, doi:10.1016/j.jmr.2005.08.013.
